# Catarrh

**Published:** 1873-03

**Authors:** 


### CATARRH.

The country is flooded with newspaper "cures" for catarrh, for pay and without pay. Some persons claim to be cured by snuffing salt water up the nose; others seem to have been benefited more by the use of warm or tepid water. The symptom of a disease is that which indicates its presence and is always curative or leads to a cure, tends to cure, or to point out the remedy. Catarrh is literally a "flowing from." Catarrh implies now that something is the matter with the nose, although there may be a catarrh of any other part of the body. As applied to the nose, it means an unusual discharge of water, or mucus. It is always the result of the irritation or inflammation of the part as a result of a cold "settling" in the nose or head, or of some disturbing cause; some persons have all the symptoms of a catarrh, if they are in a room where Ipecac is handled; the eyes water, and the nose runs profusely in a few moments. But, ordinarily speaking, catarrh is the immediate result of an inflammation of the mucous membrane or lining of the nostrils, which inflammation is caused by a slight cold.

The excess of fluid is the result of nature's effort to throw the disease out of the system; hence, to use any means to arrest this flow, is to thwart nature; if this flow is arrested in that direction, then nature endeavors to dispose of it in some other way. There is a surplus in the system; it is not good practice to add to that surplus, by eating or drinking anything; it is a cold, and the man who ceases to eat and drink anything whatever, the moment he finds he has taken a cold, will always arrest it, cut it short, and cure it within twenty-four hours, especially if he takes something to act freely on the bowels.

If, then, it is wished to arrest a discharge from the nose, cease at the same time to eat and drink anything, then you do not add to the surplus, and then, by causing the bowels to act freely, this surplus is carried off in another way.

There has perhaps never been a case of catarrh lasting for weeks and months, which has not been accompanied with one or more, or all of the following symptoms: costiveness, cold feet, dyspeptia or bad blood; in either case the only wise plan is to improve the general health, avoid taking cold, and let the nose alone, for the more it "runs" the better, as nature is thereby ridding herself of ill humors which, if allowed to remain, will poison the system and make the whole mass of blood impure.

